# A Neuroanatomical Substrate Linking Perceptual Stability to Cognitive Rigidity in Autism

**DOI:** 10.1523/JNEUROSCI.2831-18.2019

**Published:** 2019-08-14

**Authors:** Takamitsu Watanabe, Rebecca P. Lawson, Ylva S.E. Walldén, Geraint Rees

**Affiliations:** ^1^Institute of Cognitive Neuroscience, University College London, London WC1N 3AZ, United Kingdom,; ^2^RIKEN Center for Brain Science, Saitama 351-0198, Japan,; ^3^Wellcome Centre for Human Neuroimaging, University College London, London WC1N 3BG, United Kingdom, and; ^4^Department of Psychology, University of Cambridge, Cambridge CB2 3EB, United Kingdom

**Keywords:** autism, cognitive inflexibility, grey matter volume, perceptual symptom

## Abstract

Overly stable visual perception seen in individuals with autism spectrum disorder (ASD) is related to higher-order core symptoms of the condition. However, the neural basis by which these seemingly different symptoms are simultaneously observed in individuals with ASD remains unclear. Here, we aimed to identify such a neuroanatomical substrate linking perceptual stability to autistic cognitive rigidity, a part of core restricted, repetitive behaviors (RRBs). First, using a bistable visual perception test, we measured the perceptual stability of 22 high-functioning adults with ASD and 22 age-, IQ-, and sex-matched typically developing human individuals and confirmed overstable visual perception in autism. Next, using a spontaneous task-switching (TS) test, we showed that the individuals with ASD were more likely to repeat the same task voluntarily and spontaneously, and such rigid TS behavior was associated with the severity of their RRB symptoms. We then compared these perceptual and cognitive behaviors and found a significant correlation between them for individuals with ASD. Finally, we found that this behavioral link was supported by a smaller gray matter volume (GMV) of the posterior superior parietal lobule (pSPL) in individuals with ASD. Moreover, this smaller GMV in the pSPL was also associated with the RRB symptoms and replicated in two independent datasets. Our findings suggest that the pSPL could be one of the neuroanatomical mediators of cognitive and perceptual inflexibility in autism, which could help a unified biological understanding of the mechanisms underpinning diverse symptoms of this developmental disorder.

**SIGNIFICANCE STATEMENT** Behavioral studies show perceptual overstability in autism spectrum disorder (ASD). However, the neural mechanisms by which such sensory symptoms can coexist and often correlate with seemingly separate core symptoms remain unknown. Here, we have identified such a key neuroanatomical substrate. We have revealed that overstable sensory perception of individuals with ASD is linked with their cognitive rigidity, a part of core restricted, repetitive behavior symptoms, and such a behavioral link is underpinned by a smaller gray matter volume in the posterior superior parietal lobule in autism. These findings uncover a key neuroanatomical mediator of autistic perceptual and cognitive inflexibility and would ignite future studies on how the core symptoms of ASD interact with its unique sensory perception.

## Introduction

Studies of autism spectrum disorder (ASD) have mainly investigated two core aspects of the condition: sociocommunicational difficulties and restricted, repetitive behaviors (RRBs) (American Psychiatric Association, 2013). In addition to this trend, behavioral and clinical studies in the last decade have accumulated evidence for connections between these core symptoms of autism and its unique sensory perception (Robertson and Baron-Cohen, 2017): the core symptoms of ASD are correlated with hypersensitivity (Boyd et al., 2009, 2010), overstability (Robertson et al., 2013; Freyberg et al., 2015; Robertson et al., 2016), and prediction error (Lawson et al., 2017) in visual perception. Moreover, the core symptoms are associated with overevaluation of auditory information (Lawson et al., 2017) and unique interpretation of smell (Endevelt-Shapira et al., 2018).

Little is known, however, about the neural substrates underpinning the coexistence of such altered sensory perception with more complex, higher-order core symptoms in individuals with ASD (Robertson and Baron-Cohen, 2017). Neuroimaging studies identified larger signal variability in the visual, auditory, and somatosensory brain areas of individuals with ASD (Dinstein et al., 2012; Haigh et al., 2015), and the disruption of GABAergic inhibitory neuronal systems in their early visual cortex (Robertson et al., 2016). However, these studies did not report associations between such altered neural responses in the sensory areas and autistic behaviors. Another study found that ASD core symptoms are correlated with the size of population visual receptive fields in the primary visual cortex (Schwarzkopf et al., 2014), but this work did not identify relationships between the neural architecture in autism and its sensory symptoms. Although a recent population-based genetics study found genes that were commonly related to core autistic traits and unique perceptual sensitivity in typically developing (TD) individuals (Taylor et al., 2018), this research neither directly examined these genetic observations in cohorts with ASD nor investigated neural mechanisms involving the overlapping genetic patterns. Currently, therefore, the neurobiological bases that link sensory symptoms to core symptoms in autism remain unclear (Robertson and Baron-Cohen, 2017).

Here, we aimed to identify such a biological link by focusing on overly stable visual perception (Robertson et al., 2013; Freyberg et al., 2015; Robertson et al., 2016) and cognitive rigidity, an aspect of the RRB symptoms (Lopez et al., 2005; D'Cruz et al., 2013; Dajani and Uddin, 2015). We chose these perceptual and cognitive tendencies in ASD because our previous studies imply that both of them could be supported by overly stable brain dynamics in autism: although it has not been confirmed in ASD, perceptual stability during the bistable perception was predicted by less frequent transitions of large-scale brain activity patterns in TD individuals (Watanabe et al., 2014a); likewise, the unique cognitive skills of high-functioning adults with ASD were explained by overly stable transitory brain dynamics (Watanabe and Rees, 2017). Based on these observations, we hypothesized that the stable visual perception in ASD and its rigid cognitive styles should share neuroanatomical substrates.

We examined this hypothesis by searching for neuroanatomical features of cortical gray matter that were correlated with both the perceptual stability and the cognitive rigidity in high-functioning adults with ASD.

## Materials and Methods

### 

#### 

##### Overall study design.

**Figure 1.**
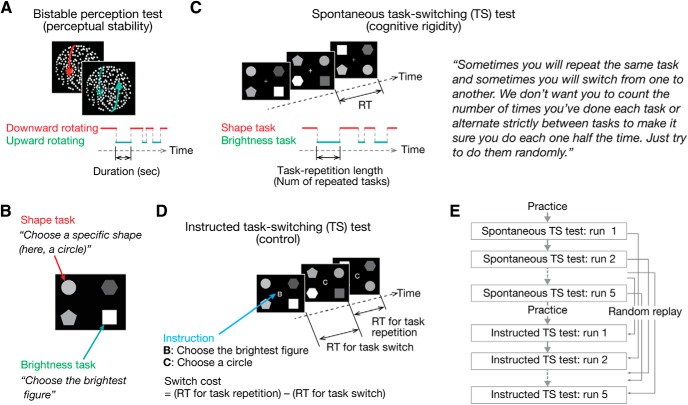
Experimental design. ***A***, In a bistable visual perception test, participants were presented with a structure-from-motion stimulus to both their eyes and asked to report when their perception switched between downward and upward rotating with a button press. ***B***, TS tests consisted of shape and brightness tasks. On each trial, participants had to choose the circle (shape task) or the brightest figure (brightness task). ***C***, In the spontaneous TS test, participants could freely choose which task to perform for each trial. We quantified cognitive rigidity as how long participants repeated the same task continuously. ***D***, In the instructed TS test, participants were given instructions about which task to perform for each trial. ***E***, Order of the instruction in the instructed TS tests was determined based on their own responses during the spontaneous TS tests. Therefore, the participants performed the same TS pattern in the two TS tests.

First, we behaviorally compared the stability of visual perception and cognitive rigidity. The stability of visual perception was measured in a test of bistable visual perception with a structure-from-motion stimulus (Kanai et al., 2010, 2011; Watanabe et al., 2014a; Megumi et al., 2015) (Fig. 1*A*). We used this stimulus because it contains no explicit social/biological information, which enabled us to evaluate perception with the least effects of the social symptoms of ASD.

Cognitive rigidity was evaluated by observing spontaneous task switching (TS) between two cognitive tasks (shape and brightness tasks; Fig. 1*B*). As in previous studies (Arrington and Logan, 2004; Poljac et al., 2012), for each trial, participants were asked to freely choose one of the two tasks and to conduct the task accurately and quickly (Fig. 1*C*). We counted how many times participants repeated the same task and used the task repetition length as an index of cognitive rigidity.

As a control, the participants also performed instructed TS tests, in which they were given explicit instructions indicating which of the two tasks they had to conduct on each trial (Fig. 1*D*). The order of instructions (i.e., the task order) was determined by the record of that participant's behavior in the previous spontaneous TS tests, so that the participants had seemingly the same task-switching (TS) experience in both the instructed and spontaneous TS tests (Fig. 1*E*). This experimental paradigm allowed us to determine whether the cognitive rigidity seen in the spontaneous TS test could be simply explained by basic cognitive/sensorimotor skills seen in the instructed TS test.

**Figure 2.**
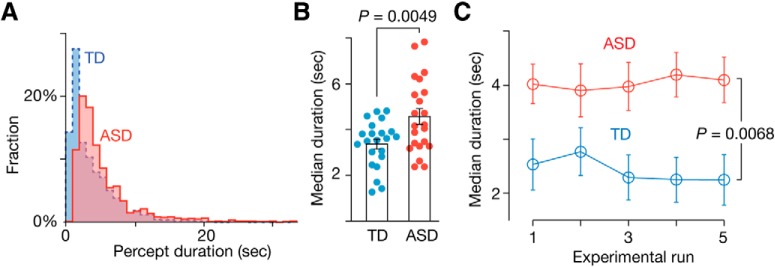
Behavior in the bistable visual perception test. Both the ASD and TD groups showed a skewed distribution of percept duration in a bistable perception test (***A***). The median duration was longer in the ASD group (***B***), which was observed throughout the experiment (***C***). Error bars indicate SEM.

**Figure 3.**
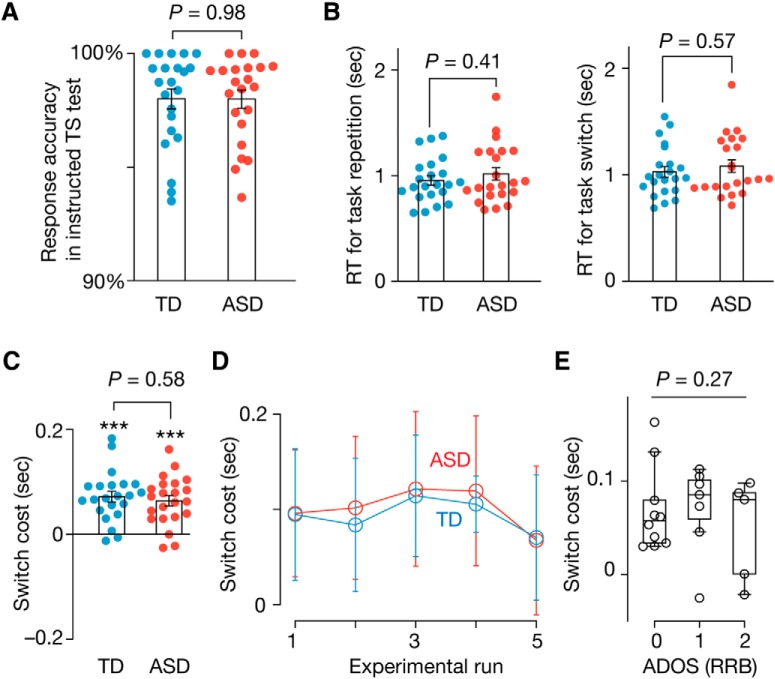
Behavior in instructed TS test. In the instructed TS test, both groups showed similar behavior. The ASD and TD groups responded accurately (***A***) with almost the same RT (***B***). The switch cost was significantly larger than zero in both the groups (*p* < 10^−5^ in one-sample *t* tests; *** in ***C***) with no significant difference between the cohorts (*p* = 0.58 in a two-sample *t* test; ***C***) throughout the test (***D***). The switch cost of the individuals with ASD was not related to the severity of their RRB symptoms (*p* = 0.27 for the main effect of ADOS RRB scores in a one-way ANOVA; ***E***). Error bars indicate SEM.

**Figure 4.**
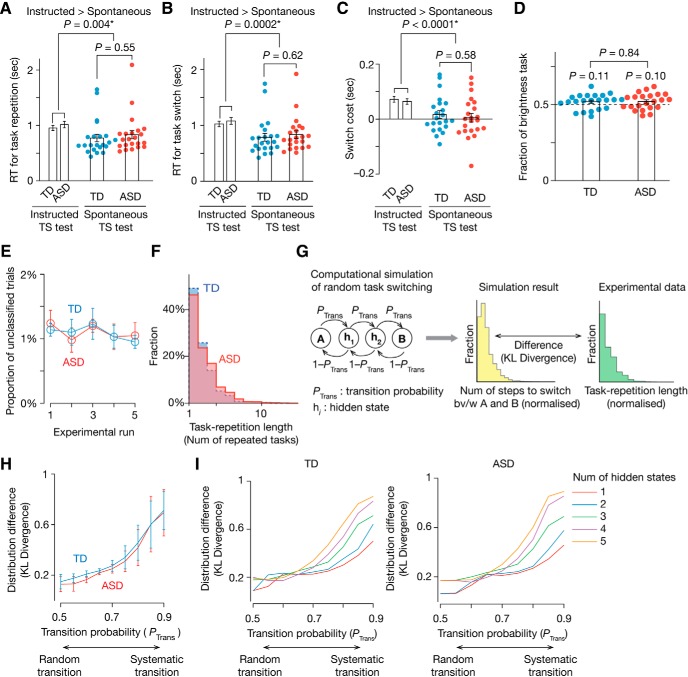
Validation of spontaneous TS test. ***A***–***C***, In the spontaneous TS test, ASD and TD participants repeated the tasks more quickly (***A***) and switched them more smoothly (***B***) than in the instructed TS test. The switch cost was not significantly different from zero and significantly less than in the instructed TS test (***C***). These results suggest that the participants repeated and switched the tasks spontaneously. *P_Bonferroni_ < 0.05. ***D***, The ASD and TD participants performed the two types of task at almost equal frequencies. Error bars indicate SEM. ***E***, The mean proportion of the unclassified trials in the spontaneous TS test was <1.5% throughout the test and not different between the ASD and TD groups. Error bars indicate SD. ***F***–***I***, In both the participant groups, the task repetition length showed a skewed, long-tailed distribution (***F***). To evaluate the randomness of the task switch, we compared the distribution with that seen in one-dimensional Markov-chain random walk simulations (***G***). When the simulation adopted more random transition probability (e.g., *P*_Trans_ = 0.5), the difference between the distributions (KL divergence) became smaller (***H***). The lines in the ***H*** show the mean KL divergence across different numbers of the hidden states varying between 1 and 5 shown in the ***I***. Error bars indicate SEM.

**Figure 5.**
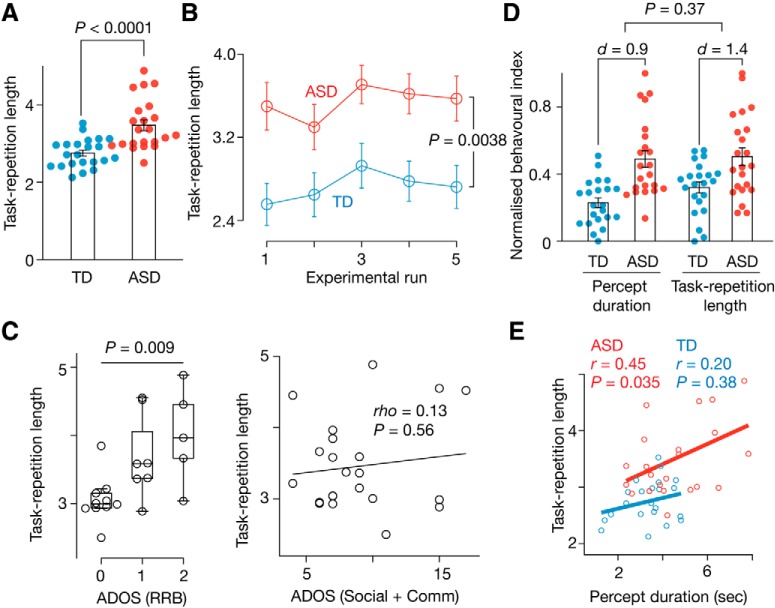
Associations between perceptual stability and cognitive rigidity. ***A***–***C***, The task repetition length in the spontaneous TS test was significantly larger in the ASD group (***A***), which was consistently seen throughout the test (***B***). In the individuals with ASD, the task repetition length was specifically related to the severity of the RRB symptoms (***C***). In ***A*** and ***C***, the *y*-axis shows the average of the median task repetition length across the five experimental runs for each participant. In ***B***, the *y*-axis represents the mean of the median task repetition length across participants. ***D***, ***E***, We compared the perceptual stability with the cognitive rigidity in the ASD group. The deviations from the neurotypical responses were almost the same in the two types of behavioral indices (***D***), and these two indices were correlated with each other in the ASD group (***E***). *d*, Cohen's effect size. Error bars indicate SEM.

Using these behavioral data, we first quantified perceptual stability in ASD (Fig. 2) and the basic skills for these TS behaviors (Fig. 3). Then, we examined the randomness and spontaneity of participants' responses during the spontaneous TS tests (Fig. 4) and investigated associations between perceptual and cognitive inflexibility (Fig. 5).

**Figure 6.**
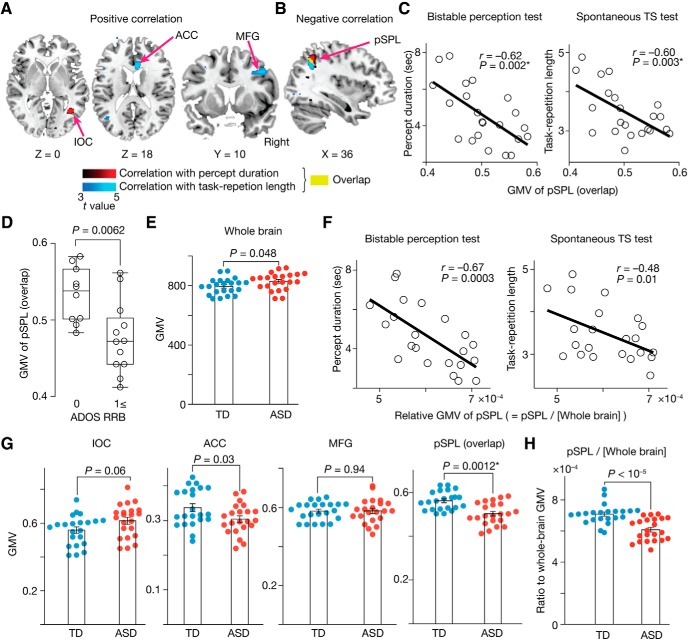
Neuroanatomical results. ***A***, ***B***, Red areas indicate voxels with GMVs that were significantly correlated with the median percept duration in the bistable perception test; blue areas indicate those with GMVs that were significantly correlated with the median task repetition length in the spontaneous TS test. The yellow area was an overlap between the red and blue regions (center, [36, −53, 44] in MNI coordinates, pSPL). For presentation purposes, the statistic brain maps adopted *t* = 3.0 as their thresholds. See also Table 3 for details. ***C***, The GMV of the overlapping pSPL region was negatively correlated with both the percept duration and task repetition length. ***D***, The GMV of the overlapping pSPL was significantly smaller in ASD individuals with severer RRB symptoms. ***E***, The whole-brain GMV was marginally larger in the ASD group. Error bars indicate SEM. ***F***, The relative GMV of the pSPL, a ratio of the GMV of the pSPL to the whole-brain GMV, still shows significant negative correlations with the median percept duration in the bistable perception test (left) and the median task repetition length in the spontaneous TS test (right). Each circle represents each individual with ASD, ***G***, Among the four regions, the pSPL was the only region with a GMV that was significantly different between the ASD and TD groups. Error bars indicate SEM. *P_Bonferroni_ < 0.05. ***H***, The relative GMV of the pSPL was significantly smaller in the individuals with ASD compared with the controls. Error bars indicate SEM.

**Figure 7.**
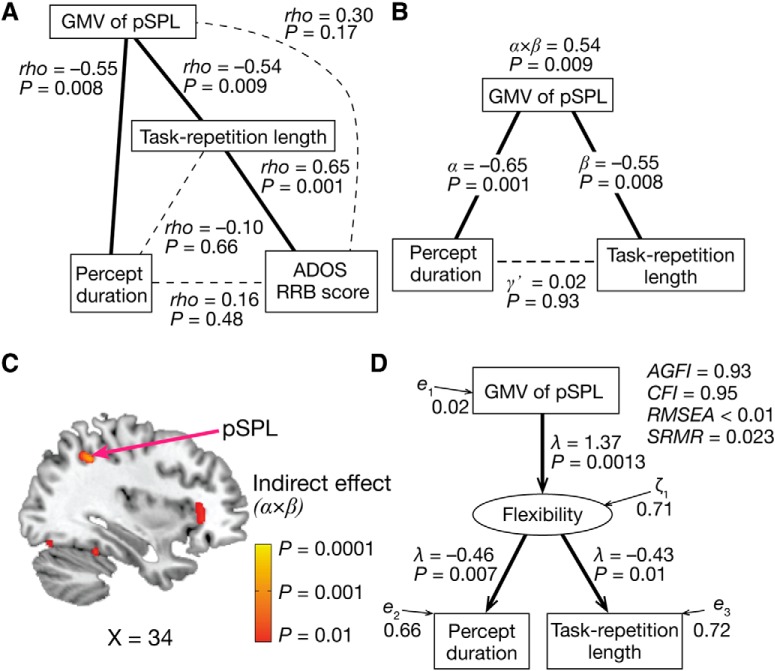
Brain–behavior associations. ***A***, Partial correlation analysis implies that the smaller GMV of the pSPL in autism linked the perceptual overstability to the cognitive rigidity and, consequently, RRB symptoms. ***B***, A nonparametric mediation analysis suggests that the smaller GMV of the pSPL would be a mediator linking perceptual stability to cognitive rigidity. This analysis used the median percept duration, GMV of the pSPL, and median task repetition length as an independent variable, mediator variable, and dependent variable, respectively. α indicates a regression coefficient of the percept duration on the GMV of the pSPL, and β denotes that of the GMV of the pSPL on the task repetition length. γ represents the direct effect of the percept duration on the task repetition length. α × β indicates the indirect effect of the percept duration on the task repetition length via the pSPL. ***C***, Colored clusters are brain regions with GMVs that had significant indirect effects (α × β) in a whole-brain nonparametric mediation analysis with the same independent and dependent variables as in ***B***. For presentation purposes, the statistic brain map adopted *p* = 0.01 as its threshold. A significant cluster was found in the pSPL (*p* = 0.0003 in [34, −52, 44] in MNI coordinates). ***D***, A structural equation modeling analysis, in which domain general flexibility was introduced as a latent variable, indicates that the pSPL is related to domain general behavioral/mental flexibility and that the smaller GMV of the pSPL in autism would attenuate such flexibility and induce perceptual overstability and cognitive rigidity. Note that the arrows did not indicate causal relationships between the variables. λ, path coefficients; *e* and ζ, residual terms.

**Figure 8.**
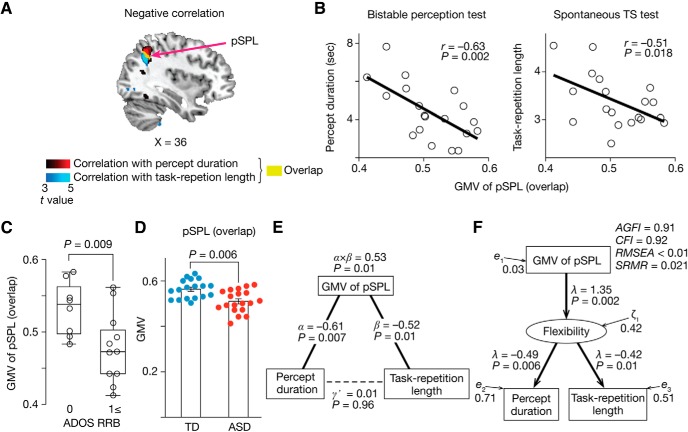
Neuroanatomical results based on male data. We repeated the neuroanatomical analyses using only the data recorded from the males with ASD (*n* = 19) and the male controls (*n* = 18). ***A***, ***B***, In the VBM, we found almost the same pSPL region (yellow area in ***A***) with a GMV that was inversely correlated with both the percept duration and task repetition length (***B***). ***C***, The GMV of this pSPL was larger in the ASD individuals with smaller RRB scores compared with those with larger RRB score. ***D***, The GMV of the pSPL area was smaller in the ASD group than in the TD group. ***E***, A nonparametric mediation analysis showed that the pSPL is a mediator linking the percept duration to the task repetition length. For the details of the analysis and abbreviations, see the legend for Figure 7*B*. ***F***, A structural equation modeling analysis indicates that the pSPL area could be related to domain general behavioral/mental flexibility. For the details of the analysis and abbreviations, see the legend for Figure 7*D*.

**Figure 9.**
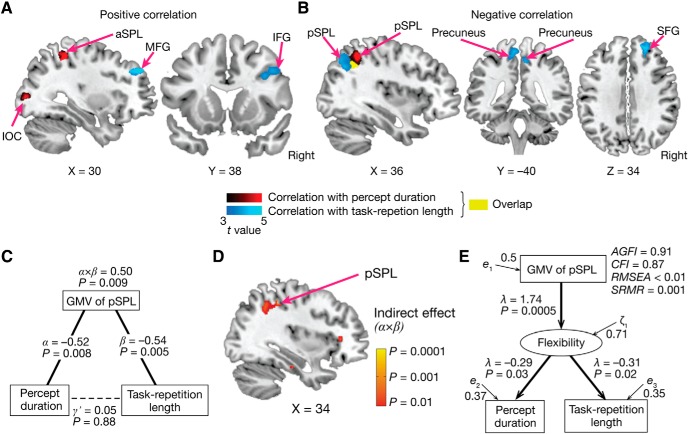
Neuroanatomical results based on TD data. ***A***, ***B***, The GMVs in the red areas were correlated with the median percept duration in the bistable perception test, whereas those in the blue areas were correlated with the median task repetition length in the spontaneous TS test. The yellow area was an overlap between the red and blue clusters. The overlapping area (***B***) was located around [34, −46, 40] in MNI coordinates. For presentation purpose, the statistic brain maps adopted *t* = 3.0 as their thresholds. aSPL/pSPL, anterior/posterior superior parietal lobule; SFG, superior frontal gyrus. See also Table 4 for details. ***C***, A nonparametric mediation analysis suggests that, even in TD individuals, the pSPL is a mediator linking perceptual stability to cognitive rigidity. For the details of the analysis and abbreviations, see the legend for Figure 7*B*. ***D***, Colored clusters are brain regions with GMVs that had significant indirect effects (α×β) in a whole-brain nonparametric mediation analysis. For presentation purposes, the statistic brain map adopted *p* = 0.01 as its threshold. A significant cluster was found in the pSPL (*p* = 0.0008 in [36, −52, 38] in MNI coordinates). ***E***, A structural equation modeling analysis indicates that the pSPL could be related to domain general behavioral/mental flexibility even in TD individuals. For the details of the analysis and abbreviations, see the legend for Figure 7***D***.

By combining these behavioral observations with structural MRI data, we conducted a voxel-based morphometry (VBM) and searched for brain areas with cortical gray matter volumes (GMVs) that were commonly correlated with perceptual stability and with cognitive rigidity in individuals with autism (Fig. 6, 7, 8) and controls (Fig. 9).

**Figure 10.**
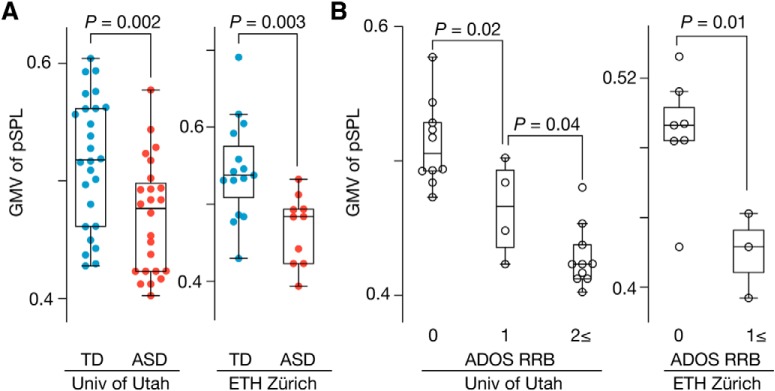
Reproducibility of smaller GMV of pSPL in autism. We examined the reproducibility of the diminished GMV seen in the pSPL in autism and its link with the RRB symptoms (Fig. 6*D*,*G*) using two independent neuroimaging datasets of high-functioning adults with ASD and age-, IQ-, and sex-matched controls (University of Utah and ETH Zürich; Table 2). In both datasets, the GMV of the pSPL (yellow area in Fig. 6*B*) was decreased in the ASD individuals (***A***) and showed smaller values when the individuals with ASD had more severe RRB symptoms (***B***).

Finally, to confirm the associations between the GMVs of the focal brain regions and cognitive rigidity in ASD, we also compared the GMVs and the severity of the RRB symptoms in our dataset and two independent neuroimaging datasets (Di Martino et al., 2014) (Fig. 10).

##### Participants.

We recruited 27 high-functioning right-handed adults with ASD and 27 age-, IQ-, and sex-matched TD individuals. This sample size was determined based on previous studies that examined perceptual stability in autism (Robertson et al., 2013, 2016). Participants were diagnosed by independent clinicians according to DSM-IV or ICD-10, and the severity of their core symptoms was assessed by a qualified administrator using ADOS (Lord et al., 1989). To reduce the heterogeneity of the participants, we focused on high-functioning adults with ASD (Full IQ/Verbal IQ/Practice IQ ≥ 85). The IQ was assessed by the Wechsler Adult Intelligence Scale (third edition, UK). We also excluded individuals with any other neuropsychiatric disorders, those whose were taking psychiatric medicines at the time of the experiments, and those who could not undergo all the experiments. In total, five ASD and five TD individuals were excluded for incompletion of the experiments due to technical problems or their visibly noisy MRI data probably due to head motion. We analyzed behavioral and MRI data of the remaining 22 ASD (three females) and 22 TD (four females) individuals (Table 1).

**Table 1.** Demographic data

	TD	ASD	*p*-value
No. of participants	22	22	—
Age (mean ± SEM)	30.8 ± 1.6	33 ± 2.0	0.41
Sex	Female: 4	Female: 3	—
Laterality	Right-handed	Right-handed	
Full IQ (mean ± SEM)	112.8 ± 3.0	119.7 ± 2.6	0.1
Verbal IQ (mean ± SEM)	114.5 ± 2.9	120.6 ± 3.1	0.1
Performance IQ (mean ± SEM)	108.0 ± 3.2	114.7 ± 2.6	0.18
ADOS Social (mean ± SEM)	—	2.7 ± 0.3	—
ADOS Communication (mean ± SEM)	—	6.2 ± 0.5	—
ADOS RRB (mean ± SEM)	—	0.8 ± 0.2	—

This study was approved by the University College London (UCL) ethics committee. All participants provided written informed consent and were financially compensated for their participation and travel expenses.

##### Test of bistable visual perception.

In the test of bistable visual perception, the participants were presented with a structure-from-motion stimulus (Fig. 1*A*), a sphere consisting of 200 moving white dots in a black background (Watanabe et al., 2014a). The dots moved sinusoidally upward and downward (angular velocity, 120°/s) with a fixation cross (0.1°×0.1°) at the center of the 21.5-inch LCD monitor (Samsung Sync Master 2233, resolution: 1680 × 1050). In each run, the participants were asked to look at this stimulus for 90 s with their chins put on a chin rest. They were instructed to push one of the three buttons according to their visual perception: one for upward rotation, another for downward rotation, and the other for unsure/mixture perception. After sufficient training sessions, the participants repeated this run five times. We conducted the stimulus presentation and response recording with PsychToolbox 3 in MATLAB (The MathWorks).

Consistent with our previous studies (Watanabe et al., 2014a; Megumi et al., 2015), mixture perception was rare in both participant groups (1.7 ± 0.3% of all stimulus presentation times, mean ± SD). Therefore, this study focused on the time during which participants had clear awareness about the direction of the rotation. For each participant, we measured the duration of such clear perception and calculated the median of the duration to evaluate their perceptual stability. We used the median duration because perceptual durations showed long-tailed distributions in both participant groups (Fig. 2*A*).

##### Spontaneous TS test.

In the spontaneous TS test (Arrington and Logan, 2004; Poljac et al., 2012), participants were presented with visual stimuli consisting of four figures with different shapes and brightness (Fig. 1*B*), and were asked to perform a shape task or a brightness task. In the shape task, they had to identify a specific shape (here, a circle), whereas in the brightness task, they were asked to identify the brightest figure. They were asked to press one of four buttons to indicate which figure in the display they chose for each task as accurately and quickly as possible. If they did not press any button in 3 s, the trial automatically ended and the next trial started.

Participants practiced the two types of task separately by repeating a 30 s trial until they could respond correctly (≥95% of accuracy) and quickly [reaction time (RT) ≤2 s]. Then, the participants underwent the spontaneous TS test consisting of 5 3 min runs.

For each trial, participants could freely choose which task they would conduct. As in previous studies (Arrington and Logan, 2004; Poljac et al., 2012), we gave them the following instructions: “You have to choose which task to perform on each trial. Ideally, you should perform each task randomly but on about half of the trials. Sometimes you will repeat the same task and sometimes you will switch from one to another. We don't want you to count the number of times you've done each task or alternate strictly between tasks to make it sure you do each one half the time. Just try to do them randomly.”

Based on the chosen figures, we retrospectively inferred the selected tasks and classified each trial into a shape or brightness trial. Participants rarely chose a figure that could not be classified into either a shape or brightness task (the proportion of such unclassifiable trials ≤1.5%; Fig. 4*E*). Therefore, we excluded such unclassifiable trials for the following analysis.

##### Instructed TS test.

In the instructed TS test, participants were given clear instructions about which task to choose. In each trial, a letter appeared at the center of the screen, and indicated the instruction. For example, “C” meant a shape task in which the participants should choose a circle, whereas “B” meant a brightness task (Fig. 1*D*). Participants were asked to perform a task according to this instruction accurately and quickly.

The sequence of the instruction letters was determined based on the behaviors in the spontaneous TS test that the participants underwent right before (Fig. 1*E*). The unclassifiable trials in the spontaneous TS task were simply omitted in the instructed TS task. Thus, the participants were supposed to have seemingly the same task repetition/switching experience as in the spontaneous TS test.

##### Behavioral analyses for TS tests.

For both the TS tests, we calculated the mean RT for task repetition, mean RT for task switching, and switch cost (mean RT for tasks switching − mean RT for task repetition) for each participant. The response accuracy was also estimated in the instructed TS test, and the median of the task repetition length was calculated in the spontaneous TS test. The mean values were used to represent the RT because RT showed normal distributions (*p* ≥ 0.24 in Shapiro–Wilk test), whereas the median values were chosen as a representative metric for the task repetition length because they showed long-tailed distributions (Fig. 4*E*). All of these behavioral indices were first estimated for each experiment and then averaged across the five runs.

We used behavioral data during the instructed TS test to evaluate sensorimotor skills to perform TS. We compared the RT for task repetition, RT for task switch, switch cost, and response accuracy between the ASD and TD groups. Then, to examine the spontaneity in the spontaneous TS test, we compared the RT for the task repetition, RT for the task switch, and switch cost between the instructed and spontaneous TS tests.

##### Validation of the randomness in the spontaneous TS test.

To examine the randomness in the spontaneous TS test, we compared the TS/repetition behaviors in the TS test with the state-switching/duration patterns in a simple random walk model.

First, we conducted a one-dimensional Markov-chain random walk simulation (Fig. 4*G*). The randomness of this simulation was iterated by changing the transition probability, *P*_Trans_, between 0.5 and 0.9 by 0.1 (*P*_Trans_ = 0.5: the most random movements, *P*_Trans_ = 0.9: the most systematic and regular movements). The number of the hidden states between the extreme states (state *h_i_* in Fig. 4*G*) was also iterated between one and five. For each parameter set, we simulated this random walk for 10^5^ steps and counted the state duration by measuring the number of the steps taken to move from one extreme to the other (e.g., movements from the state A to the state B in Fig. 4*G*). Then, we calculated the distribution of the state duration and compared it with that of the task repetition length in the spontaneous TS test. Kullback–Leibler (KL) divergence was used to quantify the difference between the two distributions. To directly compare the shape of the distributions, we normalized the distributions before assessing KL divergence.

This normalization procedure also lessened a potentially confounding effect that would be induced by the following constraint in the random walk simulation. For simplicity, we did not consider the possibility of self-recurrent movements in the random walk. This constraint would allow continuous staying in a hidden state over time, and, theoretically, the state duration in this random walk would be shorter compared with the otherwise case. However, such reduction in the state duration should be nearly cancelled out by the normalization of the resultant distribution. Thus, this random walk constraint is considered to have no significant effects on the calculation of KL divergence.

Finally, for each participant group, we examined associations between the KL divergence and the randomness of the random walk (i.e., *P*_Trans_) and identified which *P*_Trans_ could produce the most similar state duration distribution compared with that of the task repetition length.

##### Comparison of cognitive rigidity.

After confirming the behavioral randomness and spontaneity in the spontaneous TS test, we compared the median task repetition length in the test between the ASD and TD groups.

The task repetition length was sufficiently short compared with the length of one run. In every 3 min run, both the ASD and TD participants completed ≥200 trials, whereas the median task repetition length was approximately three trials in both the groups and the ASD–TD gap was less than one trial (Fig. 5*A*). Therefore, even if the ASD individuals showed longer task repetition length, they experienced task switching as often (∼65 times per run, ∼330 times per participant) as TD individuals did (∼68 times per run, ∼340 times per participant). Given this, we could assume that the longer TS length in the ASD group did not reduce the size of the behavioral data sampling and thus, did not affect the accuracy of the statistical analyses using the behavioral records of the ASD individuals.

##### Behavioral associations.

In each participant group, we calculated the Pearson correlation between the percept duration in the bistable perception test and the task repetition length in the spontaneous TS test.

We also estimated associations between these behavioral metrics and the clinical scores. For the social symptoms, we calculated correlation coefficients between the experimental indices and ADOS social and communication scores. For RRB symptoms, we compared the behavioral indices between individuals with different ADOS RRB scores using an ANOVA.

##### Neuroimaging experiment and analysis.

We obtained T1-weighted brain images using a 3 T Trio MRI scanner (Siemens Medical Systems) with the 32-channel head coil at the Wellcome Trust Centre for Neuroimaging at UCL (TR 7.92 ms, TE 2.48 ms, Flip angle 16°, spatial resolution 1 mm cubic). These MRI images were preprocessed in SPM12 in the following steps. First, the images were segmented into gray matter, white matter, and CSF in native space by the New Segment Toolbox (Ashburner and Friston, 2005). Then, the segmented gray matter images were aligned, warped to a template space (ICBM space template for European), resampled down to 1.5 mm isotropic voxels, and registered to a participant-specific template by the DARTEL Toolbox (Ashburner, 2007). Using deformation parameters calculated by the DARTEL toolbox, the gray matter images were normalized to MNI space and smoothed with a Gaussian kernel (FWHM = 8 mm). We set FWHM at 8 mm because the value was widely used (for review, see Nickl-Jockschat et al., 2012) and previous studies demonstrated that this size of spatial smoothing would improve the accuracy of VBM (Pepe et al., 2014) without increasing false-positive rates (Scarpazza et al., 2015). This preprocessing using DARTEL Toolbox included a modulation process to preserve the volume of a particular tissue within a voxel (Good et al., 2001). Thus, signal values in the preprocessed images should indicate GMVs.

We analyzed these preprocessed MRI images of the ASD group with a multiple regression model in SPM12. In VBM, we separately searched for brain regions with GMVs that were correlated with both the median percept duration and the median task repetition length. Both the regression analyses used individual ages and Full IQ scores as covariates. We then identified brain regions whose GMVs were positively/negatively correlated with each behavioral index in the ASD data (*P*_FDR_ = 0.05). The anatomical labels for the brain regions were determined based on the Automated Anatomical Labeling atlas (Tzourio-Mazoyer et al., 2002) and the Harvard–Oxford Atlas.

Next, we conducted a conjunction analysis (Nichols et al., 2005) to specify brain regions with GMVs that were correlated with both of the behavioral indices. Technically, we searched for the overlaps between the two type of statistical brain map calculated above. The statistical threshold of this conjunction analysis was equivalent or more stringent compared with those in previous studies adopting this analysis approach (Chadick and Gazzaley, 2011; Watanabe et al., 2014b).

We then investigated whether the GMV of the overlapping region (here, posterior superior parietal lobule, pSPL) was significantly different between the TD and ASD groups.

##### Brain–behavior associations.

We conducted a partial correlation analysis to examine the associations between the GMV of the pSPL, perceptual stability, cognitive rigidity, and RRB symptoms.

We then performed a nonparametric mediation analysis to test whether the pSPL was a mediator linking the perceptual overstability to cognitive rigidity in autism. The GMV of the pSPL was set as a mediator variable, whereas the median percept duration and median task repetition length were used as an independent and dependent variable, respectively. We also conducted a whole-brain voxelwise nonparametric mediation analysis using the same independent/dependent variables (*P*_FDR_ < 0.05).

Finally, we performed a structural equation modeling analysis to determine whether the GMV of the pSPL was related to domain general behavioral/mental flexibility. The model had a latent variable representing a domain general flexibility and was fitted to the behavioral and GMV data with a maximum likelihood method. As in a previous study (Schermelleh-Engel et al., 2003), goodness of fit was evaluated based on adjusted goodness of fit index (AGFI), comparative fit index (CFI), root mean square error of approximation (RMSEA), and standardized root mean square residual (SRMR).

To validate results of the above neuroanatomical analysis, we repeated the same VBM and brain–behavior analyses using data collected from only male individuals with ASD. Also, we repeated the neuroanatomical analyses using data of the TD individuals.

##### Reproducibility of GMV alteration in autism.

We examined the reproducibility of the smaller GMV of the right pSPL using two independent MRI datasets (the University of Utah and ETH Zürich) shared in ABIDE and ABIDE II (Di Martino et al., 2014) (Table 2). The participants were selected with the same criteria as in the original analysis and their T1-weighted brain images were preprocessed in the same manner as stated above.

**Table 2.** Demographic data for reproducibility tests

	TD	ASD	*p*-value
University of Utah			
No. of participants	26	24	–
Age (mean ± SD)	25.3 ± 6.3	25.3 ± 5.5	0.96
Sex	Male	Male	–
Handedness	Right	Right	
Full IQ	112.6 ± 12.0	109.9 ± 14.2	0.47
Verbal IQ	112.1 ± 12.1	107.5 ± 14.2	0.22
Performance IQ	110.3 ± 10.4	110.25 ± 16.0	0.98
ADOS Social	–	4.8 ± 2.1	–
ADOS Communication	–	7.0 ± 2.3	–
ADOS RRB	–	1.1 ± 1.1	–
ETH Zürich			
No. of participants	15	10	–
Age (mean ± SD)	23.2 ± 3.3	21.5 ± 2.7	0.2
Sex	Male	Male	–
Handedness	Right	Right	–
Full IQ	114.7 ± 9.2	108.9 ± 12.8	0.21
Verbal IQ	115 ± 12.1	109.5 ± 14.5	0.23
Performance IQ	112.7 ± 10.1	107.0 ± 11.9	0.21
ADOS Social	–	6.7 ± 1.3	–
ADOS Communication	–	2.5 ± 1.1	–
ADOS RRB	–	0.5 ± 0.97	–

We extracted the GMV of the right pSPL from each participant using the gray matter mask identified in the original analysis (yellow area in Fig. 6*B*). We then compared the GMV of the pSPL between the ASD and TD groups and investigated whether the individuals with ASD showing less GMV in the pSPL had severer RRB symptoms.

To examine the specificity of the pSPL, we conducted whole-brain voxelwise search for brain regions with GMVs that were significantly smaller in the ASD group compared with the controls (*P*_FDR_ < 0.05).

For every brain region found this VBM, we then examined associations between its GMV and the severity of RRB symptoms. First, we calculated the average GMV for each significant cluster for each individual with ASD. Next, we conducted two-sample *t* tests of the mean GMV between the individuals with ASD with larger RRB scores (ADOS RRB score ≥ 1) and the other individuals with ASD (i.e., ADOS RRB score = 0).

##### Statistics.

All *post hoc t* tests were adjusted using Bonferroni correction. We set α = 0.05/3 when comparing three behavioral indices between the two TS tests (Fig. 4*A–C*) and α = 0.05/2 when examining correlations between the task repetition length and two types of ADOS score (Fig. 5*C*) and when evaluating associations between the GMV of pSPL and two behavioral indices (Fig. 6*C*). When comparing GMV in the four different brain regions between the ASD and TD groups, we set α = 0.05/4 (Fig. 6*G*). In the whole-brain neuroimaging analyses, FDR correction was adopted. Correlations were evaluated as Spearman's coefficients when either of them was nonparametric (e.g., ADOS score). Otherwise, they were calculated as Pearson correlation coefficients.

## Results

### Perceptual stability

First, using the bistable perception test (Fig. 1*A*), we found a significantly longer percept duration in the ASD group compared with the controls (*t*_42_ = 3.0, *p* = 0.0049 in a two-sample *t* test; Fig. 2*A*,*B*). This perceptual stability in autism was consistently observed throughout the test [*F*_(1,210)_ = 7.6, *p* = 0.0068 in a repeated-measures two-way ANOVA with an (ASD/TD) × (five runs) structure; Fig. 2*C*].

### Sensorimotor skill for task switching

Next, we quantified cognitive rigidity of autism by comparing behaviors between the instructed and spontaneous TS tests (Fig. 1*B–E*).

Behaviors during the instruction-based TS test were not different between the ASD and TD groups. Both the groups accurately performed the two tasks (response accuracy > 93.5%; no group difference, *t*_42_ = 0.02, *p* = 0.98 in a two-sample *t* test; Fig. 3*A*) and showed equivalent RT for task repetition and for task switching (*t*_42_ < 0.83, *p* ≥ 0.41 in two-sample *t* tests; Fig. 3*B*). Thus, the switch cost (RT for task switching − RT for task repetition) was almost the same between the two groups (*t*_42_ = 0.56, *p* = 0.58 in a two-sample *t* test; Fig. 3*C*). This seemingly intact TS ability of the individuals with ASD did not change throughout the test (*F*_(1,210)_ = 0.041, *p* = 0.84 in a repeated-measures two-way ANOVA; Fig. 3*D*).

In addition, the switch cost, a widely used index for cognitive flexibility in TD-participant studies (Aron et al., 2004; Dajani and Uddin, 2015; Watanabe et al., 2015b), did not show significant differences between individuals with different severity of RRB symptoms (*F*_(2,19)_ = 1.4, *p* = 0.27, one-way ANOVA; Fig. 3*E*).

These results suggest that high-functioning adults with ASD had sufficient sensorimotor skills to accurately perform the two tasks and smoothly switch between them according to the instructions.

### Spontaneous and random TS

Participants experienced the same TS sequence in the spontaneous and instructed TS tests, because the timing of the switches in the instructed TS test was yoked to previous per-participant spontaneous switch timings (Fig. 1*E*). However, behavioral responses during the spontaneous TS test were distinct from those during the instructed TS test mainly in the following two points (Fig. 4), each of which indicates the spontaneity and randomness of the TS behavior, respectively.

First, the spontaneity was supported by RT analyses. Compared with the instructed TS test, the ASD and TD participants repeated tasks in the spontaneous TS test more quickly [*F*_(1,84)_ = 8.6, *p* = 0.004 in a repeated-measures two-way ANOVA with a (ASD/TD) × (spontaneous/instructed) structure; Fig. 4*A*] and switched them more smoothly (*F*_(1,84)_ = 15.0, *p* = 0.0002 in a repeated-measures two-way ANOVA; Fig. 4*B*) with significantly less switch cost (*F*_(1,84)_ = 21.0, *p* < 0.0001; Fig. 4*C*). These behaviors are reasonable if the participants switched and repeated the tasks spontaneously.

Second, the randomness was supported by the TS frequencies and intervals. The participants performed the two tasks for almost equal frequencies (Fig. 4*D*) with a small proportion of unclassified trials (mean ≤ 1.5%; Fig. 4*E*), which is reasonable if the participants switched tasks randomly. Moreover, the task repetition length showed a skewed distribution with a long tail (Fig. 4*F*), which was similar to the distribution of state-switching intervals in a random walk (Fig. 4*G*). That is, when a one-dimensional Markov-chain random walk was determined by more random transitions (e.g., *P*_Trans_ = 0.5 in Fig. 4*G*), the resultant distribution of the state-switching intervals was more similar to the actual task repetition length distribution seen in this spontaneous TS test (Fig. 4*H*,*I*).

These observations suggest that the participants performed the spontaneous TS test as spontaneously and randomly as possible.

### Cognitive rigidity

Such spontaneous TS behaviors enabled us to detect cognitive rigidity in ASD. The ASD and TD groups showed equivalent RTs for task repetition, RTs for task switching, and the switch cost (*p* > 0.55 in two-sample *t* tests; Fig. 4*A–C*), but the task repetition length was significantly longer in the ASD group (*t*_42_ = 4.5, *p* < 0.0001, *P*_Bonferroni_ < 0.05 in a two-sample *t* test; Fig. 5*A*). This was observed consistently throughout the test (*F*_(1,210)_ = 8.6, *p* = 0.0038 in a repeated-measures two-way ANOVA; Fig. 5*B*).

The prolonged task repetition length in the ASD group could not be explained by basic cognitive/sensorimotor skills: the task repetition length was not significantly correlated with the RT for task repetition, the RT for task switching, or the switch cost in either type of TS test (|*r*| < 0.19, *p* > 0.40); neither the IQ scores (Full IQ, Performance IQ, Verbal IQ) nor the age of the participants were associated with this cognitive rigidity index (|*r*| < 0.21, *p* > 0.34).

Instead, this longer task repetition in the ASD group was associated with the severity of the RRB symptoms (*F*_(2,19)_ = 6.2, *p* = 0.009 for the main effect of ADOS RRB scores in a one-way ANOVA; Fig. 5*C*), but was not correlated with the severity of the sociocommunicational symptoms (Spearman's ρ = 0.13, *p* = 0.56; Fig. 5*C*).

Together, the behavioral results from the two TS tests indicate that prolonged task repetition length in individuals with ASD is not a consequence of their sensorimotor skills or basic cognitive abilities, but is related to the RRB symptoms. This observation supports our assumption that, at least in high-functioning individuals with ASD, task repetition lengths in the spontaneous TS test are associated with their cognitive rigidity.

### Associations between perceptual and cognitive inflexibility

We then assessed links between perceptual stability and cognitive rigidity by comparing the percept duration in the bistable perception test and the task repetition length in the spontaneous TS test. We found that, in the individuals with ASD, both behavioral indices were deviated from those of TD individuals to almost the same degree (*F*_(1,84)_ = 0.82, *p* > 0.37 in a repeated-measures two-way ANOVA; Fig. 5*D*) and were significantly correlated with each other (*r* = 0.45, *p* = 0.035; Fig. 5*E*).

### Neuroanatomical substrates for perceptual and cognitive inflexibility

We then conducted a VBM and searched for a neuroanatomical substrate underlying this behavioral link between the perceptual stability and cognitive rigidity in autism (Table 3).

**Table 3.** Results of VBM using ASD data

Region	Laterality	Peak coordinate (MNI)			*t-*value
		*X*	*Y*	*Z*	
Correlation with percept duration					
Positive					
IOC	Right	40	−64	0	4.6
Negative					
pSPL/angular gyrus	Right	36	−54	46	5.1
Correlation with task repetition length					
Positive					
ACC	Right	8	22	18	4.5
MFG	Right	46	10	40	4.4
Negative					
pSPL/sLOC	Right	38	−60	42	4.8

IOC, Inferior occipital cortex; pSPL, posterior superior parietal lobule; ACC, anterior cingulate cortex; MFG, middle frontal gyrus; sLOC, superior lateral occipital cortex.

Anatomical labels were determined based on the AAL atlas.

First, we found that perceptual stability in the individuals with ASD was positively correlated with the GMV of the inferior occipital cortex (IOC) (*t* = 4.6, *P*_FDR-corrected_ < 0.05; red in Fig. 6*A*) and negatively correlated with the GMV of the pSPL (*t* = 5.1, *P*_FDR-corrected_ < 0.05; red in Fig. 6*B*).

In contrast, the cognitive rigidity in autism showed positive correlations with the GMVs of the anterior cingulate cortex (ACC) and middle frontal gyrus (MFG) (*t* ≥ 4.4, *P*_FDR_ < 0.05; red in Fig. 6*A*), and a negative correlation with the GMV of the pSPL (*t* = 4.8, *P*_FDR_ < 0.05; red in Fig. 6*B*).

That is, the pSPL was the only region whose GMV was significantly associated with both perceptual stability and cognitive rigidity in autism. In fact, the GMV in the overlapping pSPL area (yellow area in Fig. 6*B*) was correlated with both perceptual and cognitive rigidity in the ASD group (*r* ≤ –0.62, *p* ≤ 0.003, *P*_Bonferroni_ < 0.05; Fig. 6*C*). In addition, the GMV of the pSPL was smaller when the individuals with ASD had severer RRB symptoms (*t*_20_ = 3.1, *p* = 0.0062 in a two-sample *t* test; Fig. 6*D*).

These brain–behavior associations seen in the pSPL were not explained by differences in the whole-brain GMV. The whole-brain GMV in the ASD group was marginally larger compared with the TD group (*t*_42_ = 2.1, *p* = 0.048, two-sample *t* test; Fig. 6*E*), but was not correlated with either perceptual stability or cognitive rigidity (|*r*| ≤ 0.1, *p* > 0.64). Moreover, even the relative GMV of the pSPL, a ratio of the pSPL GMV to the whole-brain GMV, was still associated with both the behavioral indices (*r* ≤ −0.48, *p* ≤ 0.01; Fig. 6*F*).

We then compared GMVs of brain regions found in the above VBM (i.e., IOC, pSPL, ACC, and MFG) between the ASD and TD groups. Despite logical independence between this GMV comparison and the above VBM (Fig. 6*A*,*B*), we found that the pSPL was, again, the only region with a significant difference in the GMV (*t*_42_ = 4.2, *P*_uncorrected_ = 0.0012, *P*_Bonferroni_ < 0.05 in two-sample *t* tests; Fig. 6*G*). This difference survived even when we considered effects of the whole-brain GMV (*t*_42_ = 9.6, *p* < 10^−5^ in a two-sample *t* test; Fig. 6*H*).

Together, these findings suggest that the perceptual overstability and cognitive rigidity in autism share some neural bases and the diminished GMV of the pSPL constitutes such brain mechanisms.

### Associations among the pSPL, perceptual stability, and cognitive rigidity

Based on the above findings, we then investigated whether the pSPL was a key neural substrate linking perceptual overstability to cognitive rigidity in autism.

First, we found that this notion was consistent with results of a partial correlation analysis (Fig. 7*A*). The correlation between the percept duration and the task repetition length (Fig. 5*E*) was explainable by the associations between the GMV of the pSPL and these two autistic behaviors.

A nonparametric mediation analysis provided more direct evidence. This analysis showed that the GMV of the pSPL had a significantly large indirect effect (α × β = 0.54, *p* = 0.009; Fig. 7*B*), which suggests that the pSPL would be a mediator between the perceptual and cognitive rigidity. In addition, a whole-brain nonparametric mediation analysis demonstrated that a region in the pSPL was the only brain area that had a significant indirect effect (α × β = 0.62, *P*_uncorrected_ = 0.0003, *P_FDR_* < 0.05; Fig. 7*C*).

Furthermore, a structural equation modeling analysis indicates that the pSPL area could be related to a neural mechanism supporting domain general behavioral/mental flexibility (Fig. 7*D*).

These findings suggest that the pSPL is one of the key brain regions linking the perceptual stability to the cognitive inflexibility in autism and the smaller GMV of the pSPL would result in the two seemingly different symptoms of autism. These observations were preserved even when we conducted the analyses using only the data of the males with ASD (Fig. 8).

### Neuroanatomical results in TD individuals

We then investigated whether these brain–behavior associations observed in the pSPL were specific to individuals with ASD or they were also seen in the TD individuals.

First, we directly tested this question: we calculated the GMV–behavior correlations by applying the pSPL area determined in the above analyses of the ASD data (yellow area in Fig. 6*B*) to the TD dataset as an anatomical mask. We found that, even in the TD dataset, the GMV of this pSPL area was inversely correlated with both the percept duration (*r* = −0.42, *p* = 0.04) and the task repetition length (*r* = −0.49, *p* = 0.015).

Second, we conducted a more thorough examination by repeating the entire VBM with the TD dataset only. Compared with the observations based on the ASD data (Table 3, Fig. 6*A*,*B*), similar brain regions were associated to the perceptual stability and/or cognitive rigidity (Fig. 9*A*,*B*, Table 4; *P*_FDR_ < 0.05), and, again, the pSPL was the only region whose GMV was commonly correlated with the two behavioral indices.

**Table 4.** Results of VBM analysis using TD data

Region	Laterality	Peak coordinate (MNI)			*t*-value
		*X*	*Y*	*Z*	
Correlation with duration in bistable perception test					
Positive					
aSPL	Right	30	−40	48	4.7
IOC	Right	32	−82	10	4.3
Negative					
pSPL	Right	30	−62	52	4.9
Correlation with task repetition length in spontaneous TS test					
Positive					
MFG	Right	28	38	40	4.4
MFG	Right	42	14	34	5.1
Negative					
SFG	Right	18	40	34	4.7
Precuneus	Left	−8	−37	56	4.8
Precuneus	Right	8	−60	66	4.5
pSPL	Right	42	−62	36	4.6

aSPL, Anterior superior parietal lobule; IOC, inferior occipital cortex; pSPL, posterior superior parietal lobule; MFG, middle frontal gyrus; SFG, superior frontal gyrus.

Anatomical labels were determined based on the AAL atlas.

In fact, the GMV of the overlapping pSPL area (yellow region in Fig. 9*B*) showed significant negative correlations with both the percept duration (*r* = −0.50, *p* = 0.01) and the task repetition length (*r* = −0.54, *p* = 0.006). Moreover, as seen in the analysis of the ASD data, a nonparametric mediation analysis indicates that the pSPL area would be a mediator linking the perceptual stability to the cognitive rigidity (α×β = 0.50, *p* = 0.009; Fig. 9*C*). This pSPL area was also found in a whole-brain nonparametric mediation analysis (*P*_FDR_ < 0.05; Fig. 9*D*). Furthermore, a structural equation modeling analysis implies that the pSPL could be related to domain general behavioral/mental flexibility (Fig. 9*E*).

These results suggest that the associations between the pSPL and the perceptual/cognitive rigidity are not specific to autism and the pSPL could be one of the essential brain regions supporting human behavioral/mental flexibility.

### Replication of smaller GMV of pSPL in autism

Finally, we examined reproducibility for the smaller GMV of the pSPL in autism and its association with the RRB symptoms. This test was conducted using two independent neuroimaging datasets in ABIDE (Di Martino et al., 2014) (datasets recorded in University of Utah and ETH Zürich; Table 2).

In both datasets, the GMV of the pSPL (yellow area in Fig. 6*B*) was significantly smaller in high-functioning adults with ASD compared with demographically matched TD individuals (*p* ≤ 0.003 in two-sample *t* tests; Fig. 10*A*). Within the ASD groups, the GMV of the pSPL was decreased when the individuals with ASD had larger ADOS RRB scores (*p* ≤ 0.04 in two-sample *t* tests; Fig. 10*B*), which is consistent with the observation shown in Figure 6*D*.

In addition, this smaller GMV and association with the RRB symptoms were specific to the pSPL. In the both datasets, voxelwise neuroanatomical comparisons between the TD and ASD groups identified significant decreases in the GMV in other brain regions, such as right hippocampus and amygdala, in autism (*P*_FDR_ < 0.05; Table 5); however, none of them showed significant associations with the severity of the RRB symptoms (*t* ≤ 0.84, *p* ≥ 0.41 in two-sample *t* tests; right two columns in Table 5). Only in the pSPL was the GMV smaller in the ASD individuals with larger RRB scores (RRB ≥ 1) compared with those with smaller ones (RRB = 0) (*t* ≥ 2.7, *p* ≤ 0.022 in two-sample *t* tests).

**Table 5.** Brain regions with GMVs smaller in ASD and associated with RRB symptoms

Region	Right/left	Peak coordinate (MNI)			ASD vs TD	Association with RRB symptoms (RRB = 0 vs RRB ≥ 1)	
		*X*	*Y*	*Z*	*t*-value	*t*-value	*p*-value
Dataset collected at University of Utah							
Amygdala	Right	30	−2	−20	4.7	0.64	0.53
ACC	Right	8	44	8	4.6	−0.84	0.41
Anterior insula	Right	36	22	0	4.2	0.75	0.46
pSPL	Right	32	−54	50	4.6	2.7	0.014
Dataset collected at ETH Zürich							
Hippocampus	Right	26	−4	−22	4.2	0.39	0.71
Anterior insula	Right	36	14	−10	3.9	0.83	0.43
pSPL	Right	30	−52	58	4.1	2.8	0.022

*t*-values and *p*-values in “RRB = 0 vs RRB ≥ 1” indicate results of two-sample *t* test of GMVs between the ASD individuals with zero RRB scores and those with ≥1 RRB scores. Positive *t*-values denote that the GMVs were larger in individuals with zero RRB, whereas the negative values indicate that the GMVs were smaller.

These results add evidence for associations between the diminished GMV in the pSPL and the cognitive inflexibility in autism.

## Discussion

This case–control study has shown that overly stable visual perception of high-functioning adults with ASD is linked to their cognitive rigidity, and this behavioral link is supported by the smaller GMV of the pSPL. We first found that overstable sensory perception seen in the bistable perception test in individuals with ASD is correlated with cognitive rigidity measured by the spontaneous TS test. Then, we identified the smaller GMV in the pSPL as a neuroanatomical substrate filling the gap between these seemingly separate behaviors in individuals with ASD.

The current behavioral findings can be seen as evidence for a link between overstable visual perception of high-functioning adults with ASD and (a part of) their RRB symptoms. Multiple previous studies reported behavioral associations between autistic sensory perception and ASD core symptoms (Robertson et al., 2012; Robertson et al., 2013; Lawson et al., 2017; Endevelt-Shapira et al., 2018); however, the core symptoms examined in these earlier researches were sociocommunicational symptoms or total ASD severity, not RRB symptoms. Some behavioral studies reported an association between hyper-/hyposensitivity in visual perception and RRB in children with autism using fine clinical scales for the core symptoms such as Repetitive Behavior Scales–Revised (Boyd et al., 2009, 2010), but the relationship has not been demonstrated in an adult population. As a consequence, unlike hyper-/hyposensitivity, perceptual inflexibility has not been yet included in a subcategory of the RRB symptoms even in the latest diagnosis criteria for ASD (American Psychiatric Association, 2013).

This may be due to difficulty in finely quantifying the nonsocial features of autism in adults with ASD. In clinical settings, the RRB symptoms of adults with autism are measured on a relatively coarse scale. For example, in ADOS (Lord et al., 1989), the severity of RRB is ranked using a limited range of integers (e.g., 0, 1, or 2 in this study and 0, 1, 2, or 3 in the ABIDE datasets used here; Table 1 and Table 2). ADOS has been repeatedly validated as a tool to aid diagnosis (Lord et al., 1989) and index severity (Gotham et al., 2009; Watanabe et al., 2015a) of this autism, but such a relatively sparse scoring system of the RRB symptoms raises the possibility that this scale could not capture nuanced individual differences in cognitive rigidity. Even in behavioral experiments, autistic cognitive rigidity is known to be difficult to detect (Schmitz et al., 2006; Geurts et al., 2009; Poljac and Bekkering, 2012; Yerys et al., 2015). In particular, high-functioning adults with ASD often easily adapt themselves to instruction-based TS tests, and occasionally outperform neurotypical individuals (Poljac et al., 2010; Poljac and Bekkering, 2012). In fact, the current instructed TS test found no significant behavioral differences between the ASD and TD groups (Fig. 3*A–D*).

In contrast, the spontaneous TS test detected cognitive rigidity, a part of the RRB symptoms (Lopez et al., 2005; D'Cruz et al., 2013; Dajani and Uddin, 2015), in the high-functioning adults with ASD with a relatively large effect size (Cohen's *d* = 1.4; Fig. 5*A*,*D*). The task repetition length recorded in this TS test provided more detailed information about individual differences in cognitive rigidity and was related to the severity of the RRB symptoms (Fig. 5*C*). In addition, given that previous studies also reported unique behavioral patterns in autistic people using a similar test (Arrington and Logan, 2004; Poljac et al., 2012), this psychological paradigm appears to have a robust sensitivity to detect cognitive rigidity in autism. This improved detectability may support our ability to identify neuroanatomical bases related to cognitive rigidity in autism.

In the neuroanatomical investigation, we found that the smaller GMV in the pSPL was associated with both autistic stable perception and cognitive rigidity. In terms of the relationship between the brain area and bistable visual perception, this finding is consistent with previous reports about dissociable functions of the anterior and posterior SPLs. A series of behavioral, neuroimaging, and brain stimulation studies show that the posterior SPL destabilizes visual perception when viewing the structure-from-motion stimulus, whereas the anterior SPL stabilizes it (Kanai et al., 2010, 2011; Watanabe et al., 2014a; Megumi et al., 2015). Therefore, the smaller GMV in pSPL should stabilize visual perception, which is consistent with the current observation. The findings in this work strengthen our knowledge about the functional anatomy of the SPL and its role in bistable perception.

The association between the GMV of the pSPL and cognitive rigidity is also consistent with previous reports about neural mechanisms underlying human cognitive flexibility (Cole and Schneider, 2007; Cole et al., 2013; Watanabe and Rees, 2017). The frontoparietal network, including this region, is considered essential for flexible coordination of different types of cognitive skill (Cole and Schneider, 2007; Cole et al., 2013), and several studies reported associations between autistic cognitive inflexibility and atypical neural activity of this brain network (Mišić et al., 2015; Uddin et al., 2015; Watanabe and Rees, 2017). In addition, a meta-analysis demonstrated a critical role of the SPL in a wide range of TS behaviors (Kim et al., 2012), and another neuroimaging study using similar voluntary TS test also suggested the importance of SPL in a voluntary action (Poljac and Yeung, 2014). These previous findings are consistent with the results of the current structural equation modeling analysis (Fig. 6*G*), in which the pSPL appears to be involved in domain general mental flexibility.

This study found diminished GMV in the pSPL in high-functioning adults with ASD in three independent datasets (Figs. 6*G*, 10*A*, Table 5), which are consistent with previous findings on the neuroanatomy of autism. Although we have to carefully consider effects of the heterogeneity of ASD (Hardan et al., 2006; Salmond et al., 2007; Yu et al., 2011; Pua et al., 2017), multiple meta-analyses reported significant decreases in the GMVs of the SPL (Via et al., 2011; Duerden et al., 2012) and neighboring parietal regions (Cauda et al., 2011; Via et al., 2011; Yu et al., 2011) in individuals with ASD. The current findings can be seen as further biological evidence implying the critical role of the pSPL in autism but now linking this finding to specific behaviors.

One of the major limitations of this study is that we did not examine functional brain architecture underlying the autistic perceptual and cognitive inflexibility. Methodologically, this work focused on establishing of a psychological paradigm to detect a link between perceptual and cognitive inflexibility in autism. Based on this behavioral observation, we then searched for preliminary neuroanatomical evidence for the behavioral link. Future research should examine how the pSPL interacts (or fails to interact) with other brain areas during spontaneous task switching in individuals with ASD.

Another limitation is in the relatively small sample size used for the main experiment. The sample size was determined by previous behavioral studies on perceptual stability in autism where the number of participants was sufficient to reject the null hypothesis (Robertson et al., 2013, 2016). No previous study provided data about how strongly the spontaneous TS test can detect the cognitive rigidity in high-functioning individuals with ASD, but the effect sizes reported here provide a principle basis for determining future sample sizes necessary to identify neural bases for such cognitive rigidity.

It remains unknown how the atypical neuroanatomical development of the SPL in autism (Watanabe and Rees, 2016) is related to the development of cognitive flexibility. In addition, our findings are not necessarily directly applicable to other sensory symptoms in autism. Seemingly lower-level sensory symptoms, such as hyper-/hyposensitivity, might not involve the SPL area. For example, typical auditory perception might be underpinned by atypical neural architectures in the superior temporal sulcus. At the same time, the SPL may also play a key role in these sensory symptoms because the area is known to integrate a wide range of sensory information (Akrami et al., 2018) and be involved in the top-down allocation of attention over primary sensory regions (for review, see Moore and Zirnsak, 2017). The smaller GMV in adults with ASD reported here may affect how low-level sensory inputs are constrained by top-down expectations (Lawson et al., 2014, 2015; Palmer et al., 2017). Future studies will be necessary to clarify whether feedback processing from the SPL to sensory regions is associated with perceptual sensitivity.

This case–control study has found a behavioral association between overly stable visual perception and cognitive rigidity in high-functioning adults with ASD. Moreover, we have identified the pSPL as one of the major neuroanatomical bases supporting this behavioral association. These findings should lead to future studies on how the ASD core symptoms interact with perceptual difficulties experienced by individuals with autism, which will help us to comprehensively understand the cognitive and behavioral styles of this disorder.
